# Practical Treatise on Pneumonia, Considered as It Occurs at Different Ages, and in Its Relation to Other Diseases, Acute and Chronic

**Published:** 1842-04

**Authors:** 


### Art. VI.

*Traité Pratique de la Pneumonie aux différens ages et dans ses rapports avec les autres maladies aigues et chroniques.* Par A. Grisolle, d.m.p., Médecin du bureau central des Hôpitaux de Paris, Membre de la Société médicale d'Observation, &c. &c.—*Paris*, 1841. 8vo, pp. 747.

*Practical Treatise on Pneumonia, considered as it occurs at different ages, and in its relation to other diseases, Acute and Chronic.* By A. Grisolle, m.d. &c. &c.—*Paris*, 1841.

The name of M. Grisolle became associated with the subject of pneumonia in the year 1836, by the appearance of a short but very excellent essay on that disease in the Journal Hebdomadaire. Not satisfied with having added the analysis of original cases given in that "Memoir," to the existing stock of knowledge, M. Grisolle has, during the intervening period, steadily pursued a course of clinical investigation of the malady, examined with marked care the contributions, whether domestic or foreign, which are to be found in the French language referring to his chosen subject, and thereby produced a monograph on inflammation of the lungs possessing a character of completeness, which in the present state of knowledge could not have been given to the history of any other disease.

In conducting our readers through this bulky tome we shall assuredly not attempt to notice more than a fraction of the topics upon which enquiries are instituted by M. Grisolle; we shall not attempt to draw up a condensed history of the disease founded on his volume, but cull from the whole such facts, results, and opinions, as appear to give an additional degree of certainty to established positions, to settle points which have hitherto been subjects of debate, or direct investigation in paths promising valuable discovery to the enquirer.

The title of M. Grisolle's work attacts attention to one of its distinguishing features,—the separate consideration given to the disease as it exists in subjects of different ages, in the new-born infant, the child, the adult, and in individuals of advanced age. Such method in the treatment of the subject is in truth indispensable; so different are the conditions, both anatomical and symptomatic, observed in individuals belonging to these different categories, that descriptions, perfectly accurate if applied to patients of one age, would be seriously defective, positively and negatively, if understood to refer to those of another. In using the word age, we employ it in the sense of epoch or period of life.

The volume is mainly founded on the analysis of 373 cases collected by the author in the course of three years; during two of which he filled the office of *chef de clinique* at the Hôtel-Dieu, and during the other discharged the duties of physician in two of the largest wards in that institution. Of these 373 cases, 304 are examples of *primary* pneumonia, that is, occurring in individuals free from evident disease at the period of attack; 69 refer to subjects already labouring under some serious acute or chronic affection;—to these latter cases M. Grisolle applies the epithet *secondary*. The numerical analysis of his cases is the groundwork of all the general statements in the work; but in order to render its perusal a less arid task, he has very frequently kept back the numerical details,—preferring to make his text a sort of commentary upon these than to divert the reader's attention from the description to arrays of figures. Beyond all question M. Grisolle has thus rendered his volume less difficult of perusal; but we are disposed to think he would have acted more wisely had he, instead of suppressing such documents, collected them together in the form of an appendix.

Anatomical history and etiology. M. Grisolle follows in the steps of Laennec in recognizing *engorgement* as the first stage of pneumonia. He admits the probable correctness of Dr. Stokes's observation, that this is sometimes preceded by another condition of the pulmonary tissue, (this we formerly described,) but having himself failed in detecting it, he is unwilling to give it a place among the necessary stages of the disease. The course of the physical signs of pneumonia points to the likelihood of Dr. Stokes's observations on the point being well founded.

The weight of the healthy lung is about 235 grammes, ($7\frac{1}{2}$ oz. nearly.) M. Grisolle has found the organ when in the state of red hepatization weigh 2500 grammes, considerably more than ten times the natural amount.

The following table acquaints us with the ordinary modes of union and the relative frequency of the different stages of the disease in forty fatal cases:

| | |
|---|---|
| 1st and 2d stages united in . . . . . . | 4 cases. |
| 1st and 3d ,, . . . . . . | 3 ,, |
| 2d and 3d ,, . . . . . . | 16 ,, |
| The three stages united in . . . . . . | 2 ,, |
| The 2d stage only in . . . . . . . | 7 ,, |
| The 3d stage only in . . . . . . . | 8 ,, |

From this statement numerous very palpable inferences may be drawn.

M. Grisolle, it appears therefrom, never examined a subject in whom the disease had not already passed the stage of engorgement. We agree with him in believing, however, the malady might prove fatal at this early period of its development in cases where the entire masses of both lungs were simultaneously or almost simultaneously attacked. But such manner of seizure is singularly rare, a fact to which, as proved by M. Louis, we have had occasion more than once to call the attention of our readers.

*Site.* Many are the pages of this journal, too, in which we have been called on to consider the relative frequency of pneumonia of the right and left sides; we are tempted once again on account of the largeness of the numbers within our reach, to touch upon the point. Of 280 cases observed by M. Grisolle, 166 were on the right, 97 on the left side; in 17 both lungs were affected. Adding these numbers to those published by Messrs. Barth, Bouillaud, Briquet, Chomel, Forbes, Lombard, and Pelletan, we shall have a total of 1430 cases, of which the right lung furnished 742; the left 426; while the inflammation was double in 262. This predominance of the right over the left side exists at all ages, a fact elsewhere referred to as it respects new-born infants, (Brit. and For. Med. Rev., vol. VII. p. 78, Jan. 1839;) and established in aged subjects by the enquiries of MM. Hourmann and Dechambre. M. Lombard of Geneva, some years since, gave out a statement to the effect that pneumonia of the left side is almost twice as frequent in adult females as in males, and that the same relation of the sexes is observed in infancy, though the numerical superiority on the part of females is at that period of life not so great. The latter of these assertions does not stand examination; the only 17 cases of pneumonia in the new-born infant, in which a single lung only was affected, collected by MM. Valleix and Vernois, were every one examples of affection of the right lung. Nor is the former of them more tenable. M. Grisolle finds that pneumonia of the left side forms scarcely one fourth of the total number of cases in adult females, while it constitutes about one third in males of the same standing in point of age. The author devotes two pages to an analysis of the explanations offered by Billard, Lombard and others of the greater frequency of the malady on the right side, and having shown them to be devoid of foundation, proposes in their room the notion that it depends upon the right lung being, *from its greater size*, more exposed to the action of morbific agents. We confess this suggestion strikes us as anything but satisfactory.

As respects the frequency of double pneumonia in the adult, we believe that the results of M. Grisolle's own observation may be more implicitly trusted to than the more extensive calculation founded on the united experience of the observers named. We have ourselves very rarely met with double primary pneumonia; of 125 cases observed by M. Barth, 8 only were double; add to this the indubitable fact that the double sub-crepitant ronchus of capillary bronchitis has been, and even continues to be, mistaken for the crepitation of parenchymatous inflammation—a ready source of multiplication of cases of double pneumonia. Certain circumstances influence its development: in new-born infants it is vastly the most common form; and in advanced age, of more frequent occurrence than in adults; lobular and "metastatic" pneumonia are almost invariably double; and M. Rayer has pointed out the greater frequency with which both lungs suffer in some epidemic constitutions than in others.

Of 264 cases in which M. Grisolle was enabled to determine this point, the upper lobe was the first to suffer in 101; the inferior in 133; the middle third of the organ in 30. Like other observers who have examined this point, he finds that the tendency to disease of the upper lobe varies in different years; it has sometimes been observed to become almost epidemic. From M. Grisolle's cases it would appear that pneumonia of the apex is about two and a half times as frequent on the right as on the left side.

Since an announcement was made by M. Louis that patients observed by him with pneumonia occupying the superior portion of the lung were, on an average, aged 54, while those whose inferior lobe suffered first were only 35, age has been supposed to exercise a material influence on the primary seat of the inflammation. M. Grisolle's results would tend to confirm that of M. Louis in its general bearing; but the difference observed byhim in the ages of the two categories of patients was not by any means so great. Our author's experience confirms also the announcement of M. Briquet, that pneumonia of the upper lobes is more common in individuals of weak constitution: the connexion is not very readily seized.

M. Grisolle leaves the question of the elementary seat of pneumonic inflammation as he found it, and as it is long likely to remain, that is, undecided. How could it be otherwise when new doctrines are continually ushered into notice respecting the nature and mode of arrangement of the elementary constituents of the lungs, all of them incompatible with each other? MM. Hourmann and Déchambre have endeavoured, he observes, to explain the granular or the uniform and plane aspect of hepatization by the seat of the phlogosis: in the former case the vesicles, in the latter the inter-vesicular cellular tissue are presumed to be mainly or solely affected. Whatever be the merit and justness of this suggestion, neither belong to the writers who have just been named. The same explanation of the granular and uniform appearances is to be found in Dr. Williams's article on Pneumonia in the Cyclopædia of Practical Medicine, (p. 414,) an article not unfrequently quoted by M. Grisolle.

*Varieties.* We pass over most abundant details upon the subjects of primary, perforative, and "metastatic" abscess, and are arrested by the author's description of a lesion occurring consecutively to inflammation, and one which he considers allied to inflammation, though unprepared to pronounce an opinion upon its *exact* nature.

"In an individual who died on the ninth or tenth day of an attack of pneumonia on the right side, I found the greater part of the lung in a state of red and gray hepatization. In the centre of the lower lobe appeared a spot of four or five centimeters in extent, which was rendered very remarkable among the surrounding grayish-coloured tissue by its mahogany and blackish hues. The lung was here converted into a sort of pulp, without the admixture of clots; it *exhaled no smell*, fell away under the slight impetus given by water allowed to pour on it, and left in its place an irregular excavation, two centimeters deep, the walls of which, stained of a blackish colour, presented a great number of cellulo-vascular filaments. This morbid state had not the aspect of pulmonary apoplexy, and differed from gangrene in the absence of the characteristic odour. However, I am inclined to believe that this softening must be considered a species of gangrene of the pulmonary tissue, analogous to certain softened states of the brain and medulla."

Not having met with an example of this very peculiar lesion, we are unable to offer an opinion concerning it.

*The condition of the pleura* investing inflamed lungs furnishes a distinction between the pneumonia of infants and of aged subjects on the one hand, and of adults on the other. Concomitant pleurisy is as rare at the two extremes of life as common in individuals of ripe age.

*Dilated Bronchi.* The enquiries of Dr. Ward and of MM. Rilliet and Barthez, in contradistinction to those of Laennec, Andral, Chomel, Hourmann, and Déchambre, would seem to show that dilatation of the bronchi is a much more frequent coexisting (probably consecutive) lesion in the pneumonia of infancy than of adult and of advanced age. M. Grisolle attributes the dilatation much less to the parenchymatous inflammation than to the capillary bronchitis, which in infancy precedes or complicates the major part of pulmonary phlegmasiæ; he explains the dilatation by the eccentric pressure on the tubes resulting from the abundance of secreted fluids. M. Grisolle is, we believe, mistaken in his opinion respecting the rarity of dilatation of the bronchi in the pleuro-pneumonia of adults. Dr. Williams has, as many of our readers are doubtless aware, successfully traced the production of dilated bronchi to the influence of pleuro-pneumonia; it is true that such influence is almost peculiar to the chronic form of the disease, but in M. Grisolle's description of this form, the discussion of the topic in question is similarly omitted. We would refer M. Grisolle also to the ingenious paper of Dr. Corrigan, in the Dublin Journal of Medical Science, (vol. xvi.) on the affection which he has well described under the most ill-chosen title of "cirrhosis" of the lung.

*Cardiac concretions.* From the facts observed by M. Grisolle, the proposition originally announced by Burserius—that pneumonia is to be regarded as a cause of the formation of polypous concretions of the heart—derives strong confirmation. These facts as satisfactorily demonstrate the fallacy of the so-called *law* announced by M. Bouillaud,* that sanguineous concretions, of yellow colour, fibrinous character, and firm consistence, and interlaced with the cordæ tendineæ, constantly exist in the second stage of the malady. Had M. Bouillaud's *law* been less ambitiously stated, and coagula of all kinds, soft, black, grumous, &c. been embraced by it, it would have perhaps proved sound; though even then the number of cases on which it was founded (fourteen) and

* British and Foreign Med. Rev., vol. IX. p. 549, April, 1840.

the fact that the influence of other diseases in the same direction had not been investigated, might have fairly caused a judicious thinker to pause before he so confidently proclaimed it.

Some at least of our readers may be aware that Pasta, the great disbeliever of the theory attributing an ante-cadaveric origin to certain cardiac coagula, affirmed, that if buffy blood be received into the heart of an ox, it will form therein true "polypi." M. Grisolle performed this experiment in a modified, but certainly far from satisfactory form six times. He received blood from the veins of patients labouring under pneumonia or articular rheumatism directly into a human heart previously emptied and washed, and kept the whole in a state of perfect rest and removed from the influence of the air for twenty-four hours;—at the end of this time the blood was found forming simply black, soft coagula, scarcely adherent to the parietes. In three cases there were some fibrinous particles, either separate or mixed with the black coagulum, but in no single case those perfectly yellow, dense, elastic, closely adherent concretions, intertwined among the columnæ carneæ, met with by M. Grisolle in one fifth of the subjects dying of pneumonia examined by him. This observer shows distinctly that the inflammatory diathesis exercises the most palpable influence on the production of these latter concretions; *how*, he is unable to determine, though he inclines to believe them formed in the same manner as plastic exudations in the pleura.

*Gastritic complication.* M. Grisolle found that in one fourth of the cases examined a greater or less extent of the gastric mucous membrane was softened; and proves, from the fact of this change having occurred with equal frequency in subjects who had not and who had taken tartarized antimony, that the grounds for Broussais' anathema of that admirable salt were purely imaginary. The all-important law discovered by M. Louis respecting the development of gastric lesion in the course of febrile maladies is sufficient to account for its presence and suggest its independence of medicinal agents. Of the nature of the softening M. Grisolle is as unprepared to speak positively as his predecessors; he thinks it "probable" that he saw a case in which it depended on solution by the gastric juice. His own cases are subversive of the notion of its inflammatory character, as the characteristic conditions of phlogosis did not exhibit themselves in the vicinity of the inflamed part. Of the non-inflammatory character of the lesion under these circumstances we do not see how any doubt can reasonably be entertained.

M. Grisolle supplies some very excellent descriptive matter upon *chronic pneumonia*, both the primary species and that depending on adjacent tuberculization, (the former of which he, with the majority of observers, regards as an affection of extreme rarity,) examines with much detail the peculiarities of secondary or intercurrent pneumonia; and having thus completed the anatomical history of the disease, passes to the consideration of its etiology. We shall extract some valuable statistical information upon this branch of his subject.

*Age.* In illustration of the influence of age in the production of the disease, may be given the subjoined tables:

| Ages. | Grisolle. | Leroux, Chomel, Bouillaud, Briquet. | Totals. |
|---|---|---|---|
| 14—20 | 34 | 84 | 118 |
| 20—30 | 82 | 190 | 272 |
| 30—40 | 58 | 117 | 175 |
| 40—50 | 43 | 107 | 150 |
| 50—60 | 39 | 84 | 123 |
| 60—70 | 26 | 37 | 63 |
| 70— | 10 | 11 | 21 |
| | 292 | 630 | 922 |

The conformity of these two pretty extensive series of facts furnishes excellent motive for confiding in the general result deducible from them. But these tables do not accurately represent the influence of age on the production of pneumonia; they teach us simply the relative frequency with which the disease is observed at different ages. As M. Grisolle correctly observes, the great frequency wherewith the disease is observed in subjects between the ages of twenty and thirty may in reality depend upon the greater number living at that age. And that this is in truth the case appears from M. Grisolle's comparison of the numbers above given, with the results of the Parisian census for 1817. It appears, that although from twenty to thirty is still the period of life furnishing most cases of pneumonia, yet that the superabundance is not by any means so great as would follow from the tables. Again, the latter would show that the frequency of pneumonia between twenty and thirty is more than double that between fifty and sixty; but if the frequency be calculated according to the living population of these two decennial periods, the difference falls to about a tenth. And this difference might actually, and in all probability would, have been in the other direction in respect of individuals past sixty, had the cases appearing in M. Grisolle's tables not been observed at hospitals into which poor persons of that age are rarely known to penetrate, as there are asylums set apart for their reception. We cannot help remarking here that the calculations by which the results in the tables were corrected should have been given.

*Sex.* The greater frequency of pneumonia in males than in females has been a subject of remark among writers from a very early period: the following figures establish its degree in the Parisian hospitals for adults:

| | Males. | Females. | Totals. |
|---|---|---|---|
| Chomel | 73 | 24 | 97 |
| Briquet | 95 | 46 | 141 |
| Grisolle | 236 | 68 | 304 |
| | 404 | 138 | 542 |

Shall we from these figures conclude that the male sex constitutes in itself a predisposing cause of pneumonia? No, replies M. Grisolle: the greater frequency of the disease among males rather originates in the nature of their employments than in their sex. This judgment he bases upon the ascertained fact, that when field and other open-air labour is shared equally by men and women, the proportion of pneumonic cases among them is very closely or exactly the same. In infancy, too, females suffer as much as males: this results from the following numbers published by

| | Boys. | Girls. | Totals. |
|---|---|---|---|
| MM. Hache | 50 | 58 | 108 |
| Valleix and Vernois | 67 | 62 | 129 |
| | 117 | 120 | 237 |

In this country we have the means afforded us in our admirable system of registration of ascertaining the relative frequency of the disease in the sexes on a much larger scale. It appears that in the year 1838, out of one million living of each sex, pneumonia destroyed 1339 males and 1064 females. This result refers to all ages, all situations in life, individuals following every variety of occupation, and shows that the gross mortality of the disease still differs to a rather serious amount in the two sexes.

*Deformed chest.* Humpy subjects and those having a defective conformation of the thorax are, if the assertion of Quarin and T. Home be correct, more prone to the disease than persons with well-formed chests. M. Grisolle has put this opinion to the test of observation; and here are his results. 1. Of twenty-eight humpy persons aged from twenty to seventy-five, (the greater number had passed the thirty-fifth year,) carefully interrogated by M. Grisolle, not one had at any period of their lives suffered from pneumonia. 2. M. Bouvier, who is at the head of an orthopædic institution, has observed pneumonia but once among its inmates during the last twelve years. 3. Of a vast number of humpy subjects opened by M. Bouvier, four only presented evidence of having died from pneumonia. 4. Of ten similarly deformed subjects opened by M. Grisolle himself, one only had fallen a victim to pneumonia. These facts supply sufficient evidence of the opinion professed by Quarin and Home: but they refer to *primary* pneumonia only; *secondary* pneumonia, on the contrary, appears to be more frequent among deformed subjects than those of normally constructed chest of the same age. Thus of twenty-four humpy subjects dying of various diseases, sixteen had manifest pneumonia, and in eight of these, the pulmonary inflammation appeared the immediate cause of death. In this point of view the class of individuals referred to resembles infants aged from two to five years.

There is much minute and valuable enquiry in the pages before us on the question of the influence of one attack of pneumonia on the development of another or others; but it unfortunately will not bear condensation.

*Trades.* M. Grisolle concludes from a large body of facts, the major part of which are derived from English sources, that such trades as require great muscular efforts on the part of those following them—such, again, as are exercised in the open air and expose those engaged in them to the inclemency of the seasons, furnish about two and a half times as large a share of cases of pneumonia as persons following in-door occupations. This result, which is little more than common experience would point to, must not be received as numerically correct by any means; of this M. Grisolle is himself well aware, and accordingly pronounces a caution on the subject founded upon a more judicious estimate of the difficulties attending the investigation of the influence of profession upon disease than is common to pathological writers. He notes the infrequency of the disease among sailors, when actually on the open sea, and prevented by their situation from occasionally landing and indulging in excess of various kinds. To the regularity of life thus ensured he attributes the comparative immunity noticed. We are far, for our own parts, from contesting the reality of the prophylactic influence here recognized; but we must aver our persuasion that there is something more than this having its share in the production of the result observed. There seems in marine air to be actually something preservative from cold; at least there are few persons who have not found themselves enabled to take liberties at sea with impunity, which on land would be assuredly followed by an attack of disease. The wet decks may be paced for hours without manifest ill effects; we may envelope our limbs in sheets, not only feeling damp and clammy, but actually furnishing a fair share of moisture on pressure, and awake in the morning without the rheumatism or other miseries which would have inevitably rewarded us for the performance of such a feat on shore. Upon what this depends, it is difficult to determine; but we believe all will agree with us as to our correct general statement of the fact, and admit that regularity of habits cannot be its cause, inasmuch as persons whose mode of life had on shore been the most precisely regular are not less favoured at sea, than those who have left, with the land, the practice of drunken indulgence.

M. Grisolle finds that females following rude out-of-door occupations are more subject to pneumonia than those plying sedentary trades. He discovers no particular connexion between the function of menstruation and the outbreak of pneumonia, (his facts are very few in number however.) His enquiries prove the error of J. Frank's assertion, that women nursing are particularly liable to be seized with the disease.

*Climate and season.* From his examination of the influence of climate (which in its exact parts is founded on the statistical documents published in this country by the registrar-general and the army-board,) M. Grisolle infers that pneumonia is a disease of all countries, and everywhere, with the fewest exceptions, ranks among the severest maladies of each land. The disease is most frequent, however, in "cold countries," like England, and those in which, instance Canada, the thermometer undergoes frequent and sudden variations. For the frequency of the malady in the Bermudas and in Malta, M. Grisolle professes himself unable to account. The frequent occurrence of the disease and consequent sudden change of temperature may account for the prevalence of the disease in the latter island.*

The state of the wind at the period fifty-four of M. Grisolle's patients were attacked, was as follows:

| | |
|---|---|
| 15 North-east | 5 South-east |
| 11 North-west | 8 South-west |
| 3 North | 8 Perfect calm. |
| 4 South | |

In confirmation of the inference deducible from this table, comes the observation of MM. Hourmann and Déchambre, that during three consecutive years, whenever the number of pneumonic cases increased notably at the Salpétrière, the wind was invariably found to be from the north-east. Le Pecq had made the same remark respecting pneumonia in Normandy.

* See Sir James Clark's work on Climate, 3d Edition, p. 242.

*Sudden chill.* M. Grisolle has submitted to the most rigid scrutiny the evidence for and against the real influence of sudden chill, while the body is in a heated state, on the production of inflammation of the lungs. He well observes upon the easy sources of error in obtaining exact information respecting the extent of such influence, and points out that if we limit ourselves to the direct question—were you exposed to a sudden chill while in a heated state? we shall almost always receive an affirmative answer, for one or more of the following reasons: Because the patient mistakes the chill referred to for the rigors of invasion; because he confounds it with the greater sensibility of the body to the influence of cold, which attends the *prodrome* of acute diseases generally; and lastly, because (here is by far the most fruitful source of error,) he believes the occurrence of a chill the only way of accounting for the attack. Under these circumstances the sage clinical observer will put a series of questions, varying somewhat according to circumstances, but bearing generally upon the mode of occupation at, and the precise moment of, seizure—submit the patient to a sort of cross examination in short, whereby the truth may be elicited. Proceeding upon this plan, that taught by his illustrious master Louis, M. Grisolle found that of 205 patients, 49 only distinctly owed their malady to the sudden accession of chill. Coupled with those of two other accurate observers, these results may be considered to supply a very accurate measure of the influence under consideration: the numbers are as follows:

| | Cases. | Seizure preceded by sudden chill. |
|---|---|---|
| Chomel | 79 | 14 |
| Barth | 125 | 38 |
| Grisolle | 205 | 49 |
| | 409 | 91 |

This proportion is very different from that which the lively M. Bouillaud would lay down: regarding as useless the precautions in the manner of interrogation observed by M. Grisolle and all followers of accurate medicine, that writer, with more dogmatism than wisdom, affirms that almost every case of pneumonia, without exception, has its origin in the influence of cold. Proceeding as he does, our only astonishment is that the Broussaisist finds *any* exceptions; nor can we marvel (for it has from time to time been our fortune to follow him in his wards,) that he proclaims to be "utterly unfitted for the exercise, and more especially for the art of interrogating patients," all who presume to question the perfect accuracy of his own fancies. Such modest self-appreciation is characteristic of the man.

*Complication with other diseases.* M. Grisolle considers, in a section of much interest, the frequency of pneumonia as an intercurrent malady occurring in the course of acute or chronic diseases, as also the influence of the latter upon the development of the former. We must content ourselves with giving a mere numerical statement of some of the results he has obtained. First, be it observed, that the diseases of new-born infants are liable to be thus complicated, as, for example, hardening of the cellular tissue, and the affection termed *muguet* by the French. In children from the age of two to sixteen there are six diseases, in the course of which this disease is prone to occur in a very high proportion, as appears from the following figures:

| | | | |
|---|---|---|---|
| Secondary pneumonia occurs in | $\frac{7}{8}$ | of all cases of | croup. |
| ,, | $\frac{5}{6}$ | ,, | cancrum oris. |
| ,, | $\frac{1}{3}$ | ,, | enteritis, measles, hooping-cough. |
| ,, | $\frac{1}{4}$ | ,, | smallpox. |

The frequency of pneumonia is well known to be generally materially less in the adult affected with measles than in the infant and child; in certain epidemic constitutions, however, the proportion of pneumonic cases in adult rubeolous patients is far from small. Of 15 adults suffering from measles during an epidemic at the Salpétrière, 7 were seized with pneumonia.

M. Grisolle insists upon the frequency with which the disease complicates glanders and phlebitis. As respects puerperal fever, declared by M. Tonnellé to be thus attended in one twelfth of the cases, our author shows that a very important distinction is to be established. In that class of cases in which the anatomical lesions consist in simple metro-peritonitis with or without lymphangitis, pneumonia is so rarely developed, that out of 52 subjects dissected by M. Grisolle, not one had suffered from pulmonary inflammation, (pleurisy existed in a quarter of the cases.) But if there be uterine phlebitis, then pneumonia occurs as in all other descriptions of venous inflammation. To continue our figures: intercurrent pneumonia appeared in

| | | |
|---|---|---|
| $\frac{1}{7}$ | of all cases of | continued fever. |
| $\frac{1}{6}$ | ,, | acute affection of the brain. |
| $\frac{1}{4}$ | ,, | pulmonary tubercles. |
| $\frac{1}{4}$ | ,, | diseases of the heart. |
| About $\frac{1}{6}$ or $\frac{1}{7}$ | ,, | cancerous disease of viscera; organic disease of liver, especially cirrhosis—Bright's disease, &c. |

In the majority of cases, the development of secondary pneumonia is not explicable by any direct action exercised by the primary disease. In such cases, whether the disease be acute or chronic, there are two conditions which coexist in the majority of instances, but may exist independently of each other—these are febrile movement and weakness. To these M. Grisolle looks as the principal agents in the production of secondary pneumonia. For the influence of age, sex, &c. (points numerically considered) we must refer to the author's pages.

The simultaneous existence of bronchitis and pneumonia, the latter originally appearing as a consequence of the former, is a point which has given rise to diversity of opinion. Laennec was on the whole disposed to believe such union extremely rare; we have known an eminent auscultator affirm that he had never observed it: on the other hand it is matter of belief with some that the extension of inflammation from the lining membrane of the smaller and capillary bronchi to the air-cells is a very common phenomenon. M. Grisolle gives the result of his experience upon the subject numerically. Of 201 patients, from whom he was enabled to obtain positive and satisfactory information on the subject, seventy-six had coughed for a greater or less length of time before the development of symptoms clearly referrible to pneumonia. Of these seventy-six, twenty-three had laboured under the chronic form of bronchitis for years; in the remaining fifty-three the bronchial affection had, at the most, existed three or four weeks at the time of pneumonic seizure. The proportion of cases thus preceded seemed somewhat greater to M. Grisolle in males than in females, and was, generally speaking, greater in direct proportion to the age of the subjects; almost all the pneumonic patients aged between fifty and seventy suffered from acute or chronic bronchitis. The frequency of chronic bronchitis among subjects of this age tends to lessen very greatly the importance of and our confidence in the inference which the above facts would, on first impression, appear to warrant.

The author has found, by careful investigation, that about one quarter of pneumonic patients between the ages of sixteen and sixty have precursory symptoms; that is, functional disturbances preceding those originating in the lungs themselves. This calculation refers to primary pneumonia; such phenomena are wanting on the contrary in the greater number of cases where the disease is secondary.

SYMPTOMATOLOGY. *Pain.* We pass over the chapter upon the symptoms of invasion, and turn to that devoted to the symptoms of the established malady. Twenty-nine subjects only out of 301 escaped pain in the side, and the diagnostic value of this symptom is strengthened by the fact that it appeared in 161 out of 182 patients during the first twelve hours; seventeen of the remaining twenty-one subjects felt it before the end of the first day; the four others from the second to the fourth days. The seat of the pain in 175 cases was as follows:

| | |
|---|---|
| At the nipple . . . . . | in 89 cases. |
| At the base of the chest, about the 6th or 7th rib . . | 39 |
| Laterally at the extremity of a line perpendicular to the nipple, but 10 or 12 centimeters distant from this . . . | 13 |
| Over the entire antero-lateral surface . . . | 10 |
| Within the nipple, more or less close to the sternum . . | 6 |
| Posteriorly and especially in the infra-spinata fossa . . | 5 |
| In the axilla . . . . . . . . | 2 |
| In the supra-mammary region . . . . . | 2 |
| In one of the hypochondria . . . . . | 4 |
| In the flank . . . . . . . | 1 |
| In the loins . . . . . . . | 1 |
| In the supra-spinata fossa . . . . . | 1 |

In about one fifth of his cases M. Grisolle found that the seat of the pain corresponded precisely with that of the inflammation in the pulmonary substance; in one seventh of these the former was close to the latter; in the others the points in question were widely distant from each other. In no case however did the inflammation and pain occupy different sides of the chest; certain authors have, it is true, related cases where this amount of difference in the locality of the pain and inflammation was observed, but M. Grisolle inclines to believe that the pain may in such cases have been rheumatismal, or actually attended with subjacent pneumonia which had escaped detection.

*Dyspnœa.* Extreme acceleration of the respiration is not a fatal symptom: of two subjects in whom the number of respirations was from 75 to 80 per minute, one recovered. The following table founded on the comparison of two groups of sixteen patients, nearly alike in respect of age and the severity of their inflammation, but differing by the presence or complete absence of pain, shows that there is far from being any direct relation between the amount of local suffering and dyspnœa.

| Frequency of Respiration. | Patients with violent pain in the side. 16 | Patients having had no pain or scarcely any pain in the side. 16 |
|---|---|---|
| From 20 to 30 per minute. | 2 | 2 |
| 30 to 40 | 5 | 4 |
| 40 to 50 | 7 | 8 |
| 50 to 60 | 2 | 2 |

It has been said by MM. Bouillaud, Andral, Hourmann, and Déchambre, &c. that pneumonia of the upper rather than of the lower lobes was especially productive of intense dyspnœa. Forty-four cases, judiciously selected, from their similarity in other respects, for the purposes of comparison show, as follows, that the decision of the writers named is not perfectly just.

| Number of Respirations. | 22 Cases in pneumonia of the upper half. | 22 Cases in pneumonia of the lower half. |
|---|---|---|
| From 20 to 30 per minute. | 4 | 3 |
| 30 to 40 | 7 | 8 |
| 40 to 50 | 9 | 9 |
| 50 to 52 | 2 | 2 |

The influence so clearly supplied by this table is confirmed by the fact, that of six subjects who died under pneumonia of the upper lobes, not one had breathed more than forty-four times in a minute; whereas in four of six subjects who died with inflammation of the lower division of the organ, the respiration was from 52 to 64, in the other two 48 per minute. As a general proposition, the number of respirations was 4 or 5 in the minute greater in fatal cases than in those of recovery.

*Sputa.* Into the author's examination of the colour, viscidity, and other characters of the pneumonic sputum we need not enter, but as the characteristic expectoration of the disease appears to derive new diagnostic importance from the earliness of its appearance, as exhibited in the following table, we extract this. In 131 cases the characteristic sputa were observed for the first time:

| | | | |
|---|---|---|---|
| On the first day in | 45 cases. | On the sixth day in | 6 cases. |
| second | 31 | seventh | 5 |
| third | 14 | eighth | 2 |
| fourth | 14 | eleventh | 2 |
| fifth | 11 | twelfth | 1 |

The rusty sputum is that which is most frequently observed as the primitive form, for out of sixty-six cases there were

35 in which the sputa were at first rusty.
25 in which they were of the colour of apricots or barley-sugar.
6 in which they were sanguinolent.

In twenty cases the expectoration was perfectly white, opaque and catarrhal; fourteen of them occurred during the influenza of 1837, a proportion showing that epidemic constitution has some influence in inducing this anomalous species of expectoration.

*Dilatation of the chest.* M. Grisolle has observed two cases furnishing strong evidence, conclusive indeed, of the occurrence of dilatation of the walls of the thorax during pneumonia. One of them refers to a male aged 24, affected with inflammation of the summit of the right lung of moderate intensity. On the fifth day, when the patient was seen for the first time, the conformation of the chest was remarkable from the complete absence of supra and infra-clavicular depression on the right side; on the seventh day some advance having been made towards resolution, the hollow in both situations commenced to be more apparent, and on the ninth it was as well marked on this as on the opposite side.

AUSCULTATORY SIGNS. We have in another place (vol. IX. p. 312) adverted to the opinions held by certain observers, our author among the number, respecting the occurrence of appreciable respiratory change in pneumonia prior to the supervention of crepitant ronchus. The feebleness of respiration which we stated to constitute, according to M. Grisolle, the earliest physical sign of pneumonia, appears to have been detected in five of seven cases observed during the first few hours after the patient's seizure. It disappears rapidly, as it was only to be detected in one sixteeenth of the subjects on the second day, in the one twenty-fifth at the end of the third. He has never observed the puerile respiration announced by Dr. Stokes as the *primary* auscultatory phenomenon of the disease, but he is willing to admit that it may in some cases be the harbinger of the others, inasmuch as he has frequently detected the phenomenon in spots subsequently attacked with inflammation.

*Crepitant ronchus.* The importance of the true crepitant ronchus is now generally understood; its pathognomonic character, when distinctly developed, admitted. The precisely characteristic crepitation, however, does not always occur, a fact which cannot be too forcibly impressed on those still in their noviciate in the study of physical signs. In 34 cases observed by M. Grisolle, the primary crepitation resembled more or less the subcrepitation of capillary bronchitis. The diagnosis under such circumstances might be sufficiently obscure; in the cases referred to the presence of the peculiar sputa of pneumonia left no room for hesitation. The existence of the ronchus in a limited and circumscribed space would justly arouse suspicion as to its cause, inasmuch as the subcrepitation of capillary bronchitis exists at the bases of both lungs. This exceptional species of ronchus was chiefly observed in subjects who had passed their fiftieth year, a circumstance giving weight to the statement of Hourmann and Déchambre, respecting the extreme rarity of true crepitation in aged subjects. During the influenza of 1837, M. Grisolle observed with very great care eighteen cases of pneumonia, not one of which presented the pathognomonic form of ronchus.

*Bronchial respiration.* In the majority of cases the passage from the first to the second stage is rather sudden, and the exchange of crepitation for bronchial respiration proportionally rapid. But

"In some patients," observes M. Grisolle, "the transition from the former to the latter of these conditions is not so rapid, and there is then heard, if not in all the spots affected, at least in some of them, a peculiar sound, perfectly characteristic, and which I shall term *sarsenet sound* (bruit de taffetas.) It impresses the ear with the sensation of a piece of new sarsenet being torn; or in some instances resembles more closely the crumpling of a silk gown. This sound never exists except in the inspiration. It is often necessary to make the patient cough, or at least take a deep breath in order to make it audible. In the majority of cases the *sarsenet sound* coexists with fine dry crepitation. It may be observed in all points of the chest; however it is much the most common in the axilla, opposite the anterior border of the lung and in the outer part of the scapular fossa. I look upon this sound as a sign of hepatization, and so far as I can judge from a single fact, I believe that it characterizes induration still limited to the surface of the lung. In 1835 an old woman died in the clinical wards with pneumonia of the lower lobe of the lung; the last day of her life, I detected towards the anterior border of the axilla, and in the greater part of the supra-mammary region of the same side, a dry rough sound mingled with crepitation, and which several persons, as well as myself, compared to the crumpling of sarsenet or parchment. I still heard it distinctly when the patient was *in extremis.* The following day I examined most particularly the part of the lung where I had heard the sound and found red induration occupying a very thin stratum of the pulmonary parenchyma."

*Bronchophony.* In his observations upon the bronchophony of hepatization, the author remarks that "in some persons whose voice is naturally acute and shrill, true ægophony may be heard, without there existing any effusion: this I have chiefly ascertained in aged subjects." This we extract, not for its novelty, (we have more than once in this Journal had occasion to make a similar remark, founded on our own experience,) but because the fact is one of no mean importance, while it is one of which the beginners in auscultation seem almost universally determined to resist the cognizance.

*Puerile respiration.* M. Grisolle objects to the prevalent notion that puerile respiration is a constant phenomenon in that portion of the pulmonary substance which remains unaffected, while the share of the organ, actually diseased, is considerable. Of thirty-six subjects in whom the condition of the respiration on the healthy side was carefully noted, three only had puerile murmur; in six others this phenomenon existed on the diseased side in the immediate vicinity of the inflamed tissue, and in two of them the puerile successively passed into bronchial respiration. On the other hand this observer finds that a weakened respiratory murmur is much more frequent in the vicinity of inflamed portions of lungs. One hundred and four patients having accurately circumscribed pneumonia, were carefully examined by M. Grisolle, and in fifty of these he found the respiration more or less weakened around the diseased part. The extent of surface in which this diminution was discoverable varied; it was commonly limited to from fifteen to eighteen centimeters, but may occupy a much larger space, and even prevail in an entire lung. The sound may be somewhat less clear on percussion opposite the portion of lung affording a weakened murmur, or it may not be at all affected.

*Fremitus.* In the pages of this Journal we have already stated our want of confidence in the signs putatively deducible from the condition of the thoracic fremitus in different diseases, (vol. IX. p. 331.) In the following passage we find evidence confirmative of the justness of our scepticism: "I have," observes M. Grisolle, "studied the phenomenon of vibration of the thoracic walls in the adult subjects affected with pneumonia in its second stage; out of these ten subjects eight presented complete absence of thoracic vibration on both sides of the chest, or if it existed at all it was equally marked on the healthy and diseased sides. In two cases it appeared to me that the vibration was a little more marked opposite the hepatized parts, but the difference was inconsiderable; besides, as the disease was on the right side, it is possible this depended on a normal difference." Here the phenomenon was manifestly studied without preconceived opinion, and the result was precisely such as we affirm it will always be, when the physician divests himself of theoretical fancy, and busies himself alone with his actual perceptions. The truth is, that the mechanism of the phenomena in the healthy, as in the diseased state is but ill, if at all, understood.

We pass over a very considerable number of pages wherein the symptomatology of the disease is examined with scrupulous minuteness, and which are evidently the result of an infinite toil, because there is little in them that can be esteemed positively new. Here, nevertheless, must the physician turn, who is desirous of knowing all that can be known in the existing state of the science on the subject in question.

Crises. We turned with no small interest to M. Grisolle's chapter on crises and critical days, in the hope of finding, from the enquiries of so diligent and enlightened an observer, a satisfactory statement of the disputes to which the matter has so long given rise. He has sufficiently exposed the fallacy of the doctrine of critical days, (a fallacy we have methodically denounced in this Journal whenever an opportunity has occurred,) but leaves the question of crises very much as he found it. His result is, that in one fourth of the cases of pneumonia terminating by recovery, either at the period at which the amelioration commenced, or a little time previous to this, certain vital changes, and especially "humoral evacuations" were observable; but whether the so-called "crisis" is the cause or the effect of the change he pretends not to decide. But the truth is, the question needs not the decision of M. Grisolle.

Termination. This author is, like his predecessors, unable to indicate any trustworthy sign or signs, wherefrom the passage from the second stage of pneumonia to the third may be predicated. Upon the value of the observations of Stokes and Fournet, of which the germ may be found (as usual) in Laennec, respecting the supervention of a mucous ronchus, the impressions from his own experience appear to be rather vacillating and uncertain. Thus he tells us, "that in eight subjects in whom one lobe was in a state of complete gray hepatization without any mixture of the second stage, humid large crepitation was discovered shortly before death; but this rhonchus had existed the preceding days also, when the lung was probably only in the state of red induration. The bubbles had perhaps during the closing days acquired an increase in point of size and humidity; but these shades were too delicate to justify diagnostic inferences. Besides, similar phenomena were discovered in these subjects who died while the disease was in the second stage." This seems to settle the matter; however, M. Grisolle "admits that where a given portion of lung had for several days been the seat of blowing respiration, without mixture of crepitation, large humid crackling or actually mucous ronchus had become audible, purulent infiltration of that portion of the lung might be *suspected;* even if the blowing respiration have diminished or disappeared. *But* in order to entitle these stethoscopic characters to such diagnostic force, it is absolutely necessary that they coincide with an exasperation of the general symptoms." M. Grisolle bids fair for the fame of the Lord Eldon of auscultators. The point is an important one and is worth further investigation.

*Suppuration.* Of the almost certainly fatal termination of the disease when the third stage is established, little doubt is entertained; some believe that death is a *sine quâ non.* Yet both this opinion and the reverse are manifestly incapable of being proved so long as we are ignorant of the *signs* announcing the passage from the second to the third stage. M. Grisolle has seen these cases terminate in recovery, wherein all the *rational* signs of the suppurative process had appeared.

*Gangrene.* The extreme rarity with which gangrene of the lung originates in active inflammation of the organ, a point noticed by J. Frank, and forcibly dwelt on by Laennec, is now commonly acknowledged; yet it is not even yet sufficiently a matter of conviction with the many. The evidence furnished by M. Grisolle on this head is important. "I have had occasion," he remarks, "to see nine or ten cases of pulmonary gangrene, and in no single instance did the lesion succeed to a perfectly characterized pneumonia. Of the 305 cases of pneumonia analysed in this work, not one ended in gangrene; and of seventy cases of pulmonary gangrene recorded in the Medical Journals during the last twenty-five years, there are at the most five which can be fairly pointed to as the sequence of pneumonia." Nor is it possible to determine what may in these rare instances have been the cause of the unusual termination: to the age of the patients it cannot be ascribed, nor to the immediate cause of the disease, for both varied extremely. Nothing can be more clearly established than that the chances of gangrenous disease are not directly as the violence of the inflammation: death occurred from the seventeenth to the twenty-fourth day in the cases thus terminating—a duration indicating instead of unusual activity, a certain amount—almost of torpor, in the inflammatory process.

*Chronic pneumonia.* Yet more rare, and rarest of all, as a termination of acute pneumonia, is subsidence into the chronic state. Of such termination M. Grisolle has seen one distinct example—many practitioners pass through a long career without witnessing even one. The subject of this case had not been bled, and had partaken of a large quantity of mulled wine during the acute stage; whether the passive and active mal-treatment may have contributed to the event observed it is of course impossible to determine. M. Grisolle's experience of chronic pneumonia was too limited to allow him to give a general view of its symptomatology, and he states that his search was vain—how readily do we believe him! —for available materials in the periodical and other literature of the day.

Complications. The author's chapter on the complications of pneumonia exhibits the same evidences of patient industry as its predecessors.

*Pleurisy.* Of 247 cases of pneumonia thirty-one became complicated with pleurisy; the application of this word being restricted to cases in which the pleuritic effusion is sufficiently extensive to produce distinct physical signs. Nearly two thirds of these thirty-one individuals were from eighteen to thirty years of age, and all the others, except two, were under fifty. The quantity of fluid effused was, with a single exception in the inverse ratio of the extent of the parenchymatous inflammation; hepatized and transformed into a hard resisting mass, the lung necessarily interferes with the accumulation of fluid. And in harmony with this fact is another: in four cases the pleuritic effusion supervened, or greatly increased at the period of resolution of the pneumonia. In proportion as the fever diminished or disappeared; when the sputa ceased to be rusty; when pain was no longer felt; the physical signs, in these cases, appeared to announce extension of the disease: percussion disclosed a more extensive and complete dulness; crepitation had ceased, and was in one case replaced by bronchial respiration and bronchophony. Here is evidently a puzzling concurrence of circumstances: the abruptness with which the increase in dulness has supervened, compared with the improving condition of the local and general symptoms, furnish the only guide to correct comprehension of the case.

It is important in respect of prognosis to fix satisfactorily the justness of Laennec's notion, that the compression exercised on the lung by pleuritic fluid exercises a beneficial influence on the pulmonary inflammation, both as regards extent and degree; especially as the same doctrine is inculcated by Dr. Williams in the words, "the effusion of the pleura by its pressure modifies the effect of the inflammation of the lung." (Lectures, p. 113.) M. Grisolle's experience is completely at variance with this doctrine; "the pleural effusion appears neither to moderate nor set a limit to the parenchymatous inflammation." Still further he finds that the presence of fluid in the pleura is without influence of any notable kind on the duration of the main disease. The existence of an effusion which is developed simultaneously with the affection of the parenchyma, modifies in nowise the anatomical characters of this; it is well known that when pneumonia attacks a lung long surrounded by pleuritic effusion, the characters of the lesions are peculiar; M. Grisolle, with Laennec, describes them under the title of carnification.

*Pneumothorax.* M. Grisolle examines the cases of alleged pneumothorax supervening in the course of pneumonia, published by Drs. Graves, Hudson, and Stokes, but neglects to state the explanation of the error of those observers (in respect of the *cause* of the tympanitic sound detected under percussion), tendered some years past by Dr. Williams, and now commonly recognized as the just one. Dr. Williams has in truth shown, that the production of tracheal or amphoric sound on percussion from consolidation of the upper lobe of the lungs is extremely common. (Lectures, p. 102, &c.)

*Bronchitis.* Fifty-eight of 240 patients had distinct stethoscopic signs of bronchitis, a complication occurring with seven times greater frequency in males than females.

*Heart-disease.* Of fifty-eight patients twelve presented various morbid phenomena connected with the heart and great vessels. In three of these twelve cases the affection was pericarditis, fatal in two instances. In eight patients (exclusive of those seized with pericarditis) a modification in the quality of the sounds of the heart was detected. In two of these cases the sounds were noisy, loud, and resembling the auriculo-metallic sound; in the others obscure and stifled, without however appearing to be produced at a greater distance from the surface than usual. In one half of these cases the impulse was augmented; there was an appearance of increased size of the organ in two instances; no single subject suffered from pain at the precordial region or from palpitation. The dulness of sound existing in these cases M. Grisolle, with M. Bouillaud and others, believes dependent on the presence of coagula adhering to and interfering with the performance of the functions of the valves.

*Jaundice.* Of 277 pneumonic subjects 20 were affected with jaundice. This occurred with extreme rarity in individuals past the age of 50; was proportionally four times more frequent in males than in females; was most common in spring and summer; occurred in sixteen cases of right and only four of left pneumonia (the frequency of pneumonia generally is, however, we have seen, considerably greater on the former than on the latter side;) attended with equal frequency inflammation of the lower and upper lobes; never preceded the pulmonary disease in order of development; never acquired any very notable degree of severity; and in six sevenths of the cases in which it occurred constituted the only symptom indicative of derangement of the biliary apparatus.

*Delirium.* Uniting his own experience with that of MM. Louis, Andral, and Briquet, M. Grisolle concludes that delirium occurs in a proportion varying from one eighth to one eleventh of pneumonic cases; it is of more frequent occurrence in fatal cases than in those of recovery, and is therefore of unfavorable augury. M. Grisolle's cases, as those of MM. Andral and Briquet, show in opposition to the assertion of M. Bouillaud, that pneumonia of the summit is, to say the least, not more frequently attended with delirium than that of the base of the organ. The ill influence of double pneumonia in this direction is, however, manifest; one fourth of the subjects, both of whose lungs were involved in the disease, had delirium. In nearly one third of the cases attended by this symptom, the anatomical characters of recent inflammation of the membranes (purulent infiltration of the subarachnoid cellular tissue, &c.) were present; in the remaining two thirds the state of the brain and membranes in nowise accounted for the functional disorder. Neither was there any evident or constant relationship between the extent of pulmonary lesions and the violence of the delirium, nor between the degree of the febrile reaction and the cerebral phenomena. Hence M. Grisolle dissents from the doctrine laid down by Louis respecting the influence of febrile action in the production of the delirium of acute affections. (vid. Brit. and For. Med. Rev., vol. XII. p. 40.) He is persuaded that the idiosyncrasies of individuals, their habits and their moral condition at the period of seizure, must play an important part in its evolution, inclines to ascribe it in certain typhoid and malignant pneumoniæ to "infection" of the fluids, and believes that in many cases—here we go heartily along with him—a plausible explanation of the delirium cannot be assigned.

The various *forms* of pneumonia are considered in a learned and interesting chapter: the bilious, the typhoid, the catarrhal, the intermittent and remittent, the latent, the rheumatic, the gouty, the verminous, the puerperal and the traumatic are separately examined and most lucidly distinguished. With this announcement of the contents of this section of the work we are forced to content ourselves.

Of all acute diseases which endanger life, pneumonia is perhaps that which produces least emaciation; such is the result of M. Grisolle's observation in adults, and in persons of advanced age. The proposition will perhaps apply to children even the most youthful.

*Phthisis.* It has been laid down as a principle by MM. Barth and Roger, that if crepitant ronchus be heard at the summit of the chest, *exclusively in front,* and febrile action be present, the pneumonia thus indicated is probably tuberculous. This principle is based on the fact, that idiopathic inflammation of the superior lobe attacks the posterior much earlier than the anterior half of the organ; that, consequently, the physical signs of the disease are almost invariably first perceptible posteriorly, and when the inflammation reaches the front of the lung they continue to be more marked at its posterior aspect. M. Grisolle admits these facts, but considers them insufficient to warrant the opinion of his countrymen. The motives of his demurrer are, that he has seen two subjects in whom the stethoscopic phenomena of pneumonia were limited for several days to the subclavicular region, yet there was no evidence that these individuals were tuberculous. Besides in ten patients who presented the rational or physical signs of phthisis, and who were subsequently seized with pneumonia, this affection pursued the same course as in ordinary cases; that is to say, the auscultatory signs were first detected in the supra and infra-spinatæ fossæ, or in the axilla, and thence spread to the clavicle. Our author concludes that the position of MM. Barth and Roger would be more tenable did it simply affirm that crepitant ronchus audible at the anterior or posterior part of the summit of the lung, limited to a small space, and continuing for a long while unchanged in degree, may lead us to suspect the existence of tubercles, inasmuch as idiopathic pneumonia affects a different mode of progress and development.

DIAGNOSIS. Of the *diagnostic* importance of bronchial respiration (for we have made our way to the chapter on diagnosis), M. Grisolle professes a different opinion from that held by some of his countrymen. M. Andral, for example, affirms that the existence of blowing respiration furnishes an excellent means of distinguishing pneumonia from pleurisy. MM. Barth and Roger maintain that blowing murmurs are heard with such extreme rarity in pleurisy, that when they are discovered something more than simple pleurisy is to be thought of. M. Grisolle, on the contrary, has found blowing respiration and bronchophony in half the cases of pleurisy he has carefully observed (upwards of 50); but in one fifth only of them did these phenomena present their pneumonic characters. English observers seem to coincide with M. Grisolle. Dr. Stokes says that "bronchial respiration is by no means uncommon in pleuritic effusion, and may be observed in the most recent as well as in chronic cases." (Diseases of the Chest, p. 494.) Dr. Williams writes, "when œgophony is most distinct, it is often coupled with bronchial respiration." (Lectures, p. 89.) Dr. Walshe admits the occurrence of blowing respiration in pleurisy, but believes it comparatively uncommon, and distinguishable from its never, so far as his experience goes, acquiring the intensity of the blowing murmurs of hepatization; "it appears distant and subdued, and is besides heard over a very limited extent of surface." (Cyclopædia of Surgery, art. Empyema, p. 109.) This statement of Dr. Walshe agrees accurately with the account given by M. Grisolle of the characters of the more usual species of bronchial respiration occurring in pleuritic effusion, but seems to make no provision for the actually pneumonic condition of the phenomenon observed by the French author in one fifth of his pleuritic cases. Comparatively speaking, this difference is unimportant; the essential point is to determine whether bronchial respiration be really present in some cases or not, and the weight of testimony is evidently for the affirmative.

PROGNOSIS. The subject of prognosis is handled with much skill by

M. Grisolle; the completeness of the chapter being indicated by its being divided into nineteen heads. The influence of early or tardy administration of medical aid is better shown by the following table than it could be in any other possible way:

| | |
|---|---|
| The mortality among patients admitted the first 3 days | $\frac{1}{13}$ |
| ,, ,, ,, 4th day | $\frac{1}{8}$ |
| ,, ,, ,, 5th | $\frac{1}{6}$ |
| ,, ,, ,, 6th | $\frac{1}{4}$ |
| ,, ,, ,, 7th | $\frac{1}{3}$ |
| ,, ,, ,, 8th | $\frac{1}{2}$ |
| ,, ,, ,, 9th | $\frac{1}{3}$ |
| ,, ,, ,, 10th | $\frac{1}{3}$ |

Delay here is truly death; a fact which had been sufficiently established in this country, in France, and in the United States in respect of typhoid fever, but of which no numerical demonstration, as regards pneumonia, had hitherto appeared. The explanation of this is well and feelingly given by M. Grisolle:

"We must not here alone consider the mere absence of all active treatment, whereby the disease is allowed to follow its course unmolested, but take into account the fact that patients of the indigent classes are for the most part exposed to anti-hygienic conditions more or less baneful. Many of them continue to walk about or to work during the first days of the affection; many of them force themselves to eat without feeling the inclination; some of them, deprived of sufficient clothing or inhabiting imperfectly closed rooms, are exposed for several days to continual cold. Finally, we must not forget the fatigue endured by such poor patients as are obliged to come to the hospital on foot, from a greater or less distance, exposed to all the inclemency of a perfidious climate."

TREATMENT. Nearly 200 pages, upwards of a fourth part of the volume, is, by a singular anomaly in French medical literature, devoted to the subject of treatment. Were it only to commemorate this signal departure on the part of a French writer from established custom—a custom which we had begun to deplore as incorrigibly engrafted on the literary system of France—we should feel disposed to notice at some length M. Grisolle's therapeutical lucubrations. But the truth is, they have more solid claims upon the attention; they are the result of much observation, of close study, and patient examination of cases under every possible aspect: all this by a writer evidently free from prejudice, whose stedfast object, the attainment of truth, is stamped in legible characters on each page of his enquiry.

*Bloodletting.* Venesection naturally engages the author's attention first, and he commences with the question,

1. *Is bleeding useful in pneumonia?* In other words, is it better to bleed pneumonic patients, or to leave their malady to the natural course? a query which, as M. Grisolle observes, may to some persons appear to savour not a little of absurdity. Nevertheless, it is sufficiently obvious that unless we have ascertained the mean mortality of the disease when left to itself, we are without a standard whereby to test the real value of bleeding or of any other given mode of treatment. And it is especially necessary to raise the question at Paris, where it is notorious the late M. Biett treated every case of pneumonia that appeared in his wards during an entire year by emollient drinks and linseed poultices, and could point, it is alleged, in vindication of his *practice*, to a low rate of mortality; where, again, it is notorious that at the present hour the accomplished slayer of dogs, the Chesterfield of vivisectors, M. Magendie, does little else than rival the inaction of his late *confrère* of St. Louis. The humanity and conscience of our author made him shrink from the application of the expectant treatment in cases of any severity, but having, while engaged in these researches, met with eleven cases in which the inflammation was of very limited extent and attended with mild general reaction, he simply prescribed repose and rigid abstinence, demulcent drinks, and in some cases a gentle laxative. The patients were admitted, on an average, on the fourth day of the disease; in nine among them the lung was hepatized, in twenty the inflammation was in, and never passed beyond, the first stage. These cases are compared with a series of thirteen others, four only of which had not already reached the stage of hepatization; the general symptoms were mild, the patients still young, and they were all bled twice, locally or generally, on an average the fourth day. Now M. Grisolle does not content himself with the mere affirmation that benefit accrued from the use of the lancet, but he shows in what manner that benefit acted, and above all, its amount. We shall place beside each other, in order to render the contrast stronger, the results obtained in the two series of cases.

| 11 cases of mild pneumonia, not treated by bleeding. | 13 cases of mild pneumonia; bleeding performed on an average on the 4th day. |
|---|---|
| *The Characteristic* | *Sputa.* |
| Continued till the 9th day. | Ceased before the end of the 6th day; about 48 hours after venesection. |
| *Pain in* | *the Side.* |
| Although diminished at the end of 4 or 5 days, never disappeared before the 8th, and frequently continued to the 20th, 25th, and 27th days. Its mean duration = 15 days. | Invariably relieved by bleeding, whether this was general or local; disappeared completely on from the 2d to the 12th day, and on an average on the 8th day. |
| *Febrile* | *Reaction.* |
| Ceased about the 10th day; convalescence commenced the 11th or 12th day. | Had ceased abruptly at the end of the 7th day; then commenced convalescence, which was thoroughly established 24 hours after. |
| *Auscultatory* | *Phenomena.* |
| Commenced to decrease at the end of the 2d week, that is 4 or 5 days after the cessation of fever, but continued to be more or less manifest till from the 22d to the 30th day, that is, the lung was still more or less impermeable. | Began to decrease with the cessation of the fever, that is the 7th day, and in the subjects carefully examined till their departure from the hospital, the lung had completely recovered its permeability, on an average on the 12th day. |

We have seldom recorded the results of experience with more thorough satisfaction than we feel in committing these to our sheet; and for this simple reason, that we ourselves (and the multitudes who doubtless have acted like us) find herein not only the justification, but the demonstrated utility of the practice of bleeding (unless some peculiar contraindication exist), even in the slightest case marked by the pathognomonic rhonchus or sputa. It is scarcely necessary to declare that we were convinced of our correctness in invariably ordering loss of blood; that is, as well convinced as we could be from the general impression derived from our experience, unaided by its accurate analysis. Yet we must honestly confess that we have sometimes had our misgivings as to the wisdom of the course, and been almost inclined to yield to the pressing solicitations of patients and of relatives, that the lancet might be spared. We shall be sterner bleeders in future.

The table given at page 402, representing the relative mortality among patients admitted at different periods of the disease, seems to involve, (and does so in truth, though not to the degree appearing at first sight,) as a necessity, the utility of treatment of some kind. However, it must be remembered, as we have already had occasion to say, that those who delay their entry into hospital not only do not receive positive aid, but are frequently submitted to what may be called negative treatment of the worst kind.

2. *Should we bleed in all cases of pneumonia without exception?* It has been said, and is taught in some quarters, that venesection is dangerous at the two extremes of life, in early infancy and in extreme old age; that at these periods a collapse, from which recovery shall be impossible, is to be dreaded as the natural sequence of loss of blood. Idle apprehensions! Experience proves that not only may bleeding be performed upon both classes of subjects without risk, but with manifest advantage. If we have a settled conviction in therapeutics, it is, that the fatal issue of pneumonia may be averted even in the first week of extra-uterine existence (we have, as we believe, twice so averted it ourselves) by abstraction of blood. And is advanced age *per se* a sufficient motive for abstaining from bloodletting? Even as little as in the new-born infant. Morgagni drew blood with success from nonagenarians; five of six subjects, who had passed their seventieth year, were sufficiently vigorous to bear venesection with advantage. To insist upon such cases is needless, for they are of frequent occurrence. On the other hand, a constitution deteriorated by misery, by bad nourishment, by grief, excess, or previous disease, at whatever age it exist, furnishes a formal contraindication to bleeding. While certain epidemic constitutions prevail, it is a well-established fact that venesection is inadmissible, and this is also, with rare exceptions, the case, according to M. Grisolle, with secondary pneumonia of every species.

M. Grisolle labours to remove the popular prejudice respecting the danger of bloodletting in women upon the point of or actually menstruating. “The flow of the catamenia, no matter in what manner it be advancing, constitutes no obstacle to bloodletting, provided the existing symptoms proclaim its utility.” He cites Delamotte and J. Frank in favour of his opinion; the latter states that bleeding never suppressed either catamenia or lochia, when practised for the relief of inflammation; and M. Grisolle observes that in those pneumonic patients who had their menses while in the hospital, the discharge flowed about as abundantly and as long as in the state of health. M. Grisolle is equally strong in his assertion of the propriety of bleeding pregnant women, where symptoms require it.

3. *Should we bleed at all periods and stages of pneumonia?* M. Grisolle rightly maintains that no period of a pneumonia is too advanced for bleeding, provided the indication for loss of blood be thoroughly established. He has himself prescribed, and known M. Chomel to prescribe, venesection on the twelfth, fifteenth, and seventeenth days of the malady. But although we be justified in bleeding at any period of the disease, it is certain (as was first shown by M. Louis several years since) that the utility of the measure is much greater when it is had recourse to at an early than a late period. M. Louis found that patients bled during the first four days recover, *cæteris paribus,* four or five days earlier than those bled at a more advanced stage of the affection. The large series of cases observed in four successive years by M. Grisolle, gave a precisely similar result, even to the mean number of days gained by the early bloodletting.

M. Andral believes that the existence of the third stage of the disease does not always contraindicate the use of the lancet, inasmuch as the third often coexists with the first two stages, and these may still be counteracted by bloodletting. M. Grisolle well observes upon this, that the problem of the propriety of bleeding under these circumstances is one which in the existing state of knowledge cannot be resolved, for a reason already mentioned, our inability to diagnosticate with sufficient certainty the actual existence of the third stage. Be that as it will, such facts as the following lead M. Grisolle to hold a contrary opinion to M. Andral: three patients in whom the inflammation had certainly reached the stage of suppuration, (as proved, we presume, by post-mortem examination,) but the state of whose pulse did not precisely contraindicate bleeding, were bled to ten or twelve ounces; they fell almost immediately into a state of collapse, which terminated by death in four, six, and eight hours. Another subject who was in a complete state of collapse seemed to rally under tartar emetic and a blister; the improvement tempted the medical attendant to draw twelve ounces of blood: in three hours the patient had ceased to live.

4. *How and where should we bleed?* With the response to this query we need not detain the reader, as it presents nothing remarkable.

5. *With what frequency should bleeding be practised, and at what intervals should successive bleedings be performed?* We shall not follow M. Grisolle through his sketch of the practice of the great practitioners of the last century, and of the modern Italian school, but turn at once to the critical sifting to which he submits the notorious "*formula des saignées coup sur coup,*" which M. Bouillaud has by systematic laudation forced upon the attention of French practitioners. The treatment by repeated and large bleedings following each other at short intervals, we need scarcely say, no more *originates* with M. Bouillaud than with any practitioner in the metropolis. But upon a people who, like the good Parisians, have commonly no inkling of what is done beyond the limit of their own land, it is not a very marvellous achievement to succeed in palming any notion new to France as a thorough novelty. M. Bouillaud performed this small wonder. The new treatment of the animated professor was applauded uproariously, his inventive genius was the burden of all conversation on the benches of the Académie Royale; beardless striplings stood mute with admiration, as " one bleeding more" was ruthlessly ordered for the patient who even then seemed to have scarce a little ounce of blood in hisframe. But what matter if the patients disappeared; the raw *externe* had got his hand in with the lancet, and such was his skill at the Clinical School of La Charité, that a *saignée blanche* was there unknown. After a time, however, even Parisians discovered that their vivacious therapeutist was not an *inventor:* the more serious question, was he an *improver*, remained to be answered. Of course, according to himself he was this improver; nay more, *seipso judice*, he figured as the greatest public benefactor France had produced for heaven knows how long, as by his "formula" the lives of, we forget how many, thousand citizens would yearly be saved to the state. Sober men, however they were at first staggered by the confidence with which all this was proclaimed, soon began to desire a close examination of the evidence on which these pæans were sung, and in M. Grisolle they have found a scrutator likely to lighten the weight of M. Bouillaud's claims to the civic crown.

Suppose a case of pneumonia of moderate extent and severity in the first or, at most, the second stage in an adult of ordinary strength and constitution, the "formula" according to which M. Bouillaud would treat him is as follows:

"*First day.* Bleeding to four 'palettes'* in the morning; to three or four in the evening. Between these, thirty leeches to the side, or three 'palettes' of blood to be removed by cupping.

"*Second day.* Bleeding to four 'palettes;' if the pain in the side continue, the leeches to be repeated.

"*Third day.* If the pneumonia be still present, bleeding to the amount of three or four 'palettes' must without hesitation be performed.

"*Fourth day.* Should the disease, which is rare, not have yet yielded, bleeding may again be practised, but it is commonly better to renounce bloodletting and apply a large blister to the side.

"*Fifth and sixth days.* Nothing remains now to be done but to watch the state of the patient attentively. In the most common cases, resolution rapidly takes place, and some appetite already begins to be felt. In some exceptional cases, a sort of recrudescence of the malady may arise, and the physician may be obliged to return, but with more reserve and soberness to bloodletting. M. Bouillaud is of opinion that it is now or never that tartar emetic might be had recourse to with advantage." (Grisolle, p. 582.)

Here then M. Bouillaud measures out *à priori* the quantity of blood to be lost by every individual seized with pneumonia; the extent of the disease, observes M. Grisolle, the stamina of the subject, and other conditions which are commonly deemed of some importance, are not thought of. M. Bouillaud affirms, it is true, that on the contrary he *does* regard all circumstances which may and ought to modify the treatment; but is this affirmation justified by his practice? A man, aged 36, "worn out by saccharine diabetes," (M. Bouillaud himself speaks,) reduced to a state of considerable emaciation, was seized with adynamic pneumonia, came under the care of M. Bouillaud, and forthwith underwent the necessary depletions in the shape of these bleedings of twelve ounces each. The patient lived till the seventh day. Is this having regard to all the circumstances of individual cases? But M. Bouillaud's sole regret is that he did not bleed the patient more!

But M. Bouillaud has not confined himself to a mere general assertion of the superiority of his method of treatment; he has, upon various oc-

* The palette is equal to four ounces.

casions, published numerical statements of the results of his practice, "arranged," according to M. Grisolle, "in a manner well calculated to lead the unwary into error." A selection, for example, is made of 56 cases of pneumonia, some of them of mild character and terminating favorably under the use of emollients only, 2 only ending fatally; in other words, a mortality of 1 in 28 of those attacked. Here is very evidently a most extraordinarily low rate of mortality. But in the first place, as M. Grisolle remarks, one third of these patients recovered without M. Bouillaud's formula; 35 patients, of whom 2 died, accordingly remain for the credit of the formula: still a degree of success sufficiently remarkable. But now comes the notable part of the story. After having pompously announced a mortality of 1 in 28, M. Bouillaud finds himself obliged, at the close of the very same paper, to give as his definitive result 1 death in 8¾ cases. "Oh! what a falling off was there!" Still even this proportion of 1 in 8¾ signifies by no means a despicable share of success in the treatment of pulmonary inflammation. Unfortunately even from this less prominent vantage-ground the inexorable M. Grisolle drives his victim; he shows that even this result may be accounted for in a different way than by the efficacy of the *saignées coup sur coup.* We proceed with M. Grisolle's demonstration of this.

The last Memoir published by M. Bouillaud in the papers of the Royal Academy of Medicine (nominally by one of his pupils, M. Pelletan) refers to 75 cases, wherein are included those already referred to. Of these, 49 only were treated by the formula; 6 of the 49 died, or about 1 in 8, not 8¾, as appeared from taking all the cases indiscriminately. But this result (far from an unfavorable one) is evidently due to circumstances wholly unconnected with the treatment. 1st. It is known that pneumonia, treated in the ordinary mode, destroys scarcely one seventh of those attacked whose age is between thirty and forty. Now M. Bouillaud's patients had scarcely attained the mean age of thirty-three; what marvel, then, that one eighth only of them succumbed? The simple circumstance of the age of the patients not only suffices to explain the apparently favorable character of the results at La Charité, but accounts, according to M. Grisolle, for the "impunity with which M. Bouillaud has employed his method; the tenacity of life found in young subjects can alone have counterbalanced the evil effects of venesection carried to extremes."

And, again, M. Bouillaud has been to no small extent favoured by the unadvanced condition of the disease when his patients came under his care: in sixteen of the forty-nine subjects the affection had not passed the first stage, or had only reached the point of transition from the first to the second; in ten subjects the first two stages were combined; in thirteen only was hepatization perfectly established.

There is, further, a third and very important circumstance bearing in the same direction, connected with the conditions of M. Bouillaud's patients. Pneumonia is a much more fatal disease in females than in males;* now of the cases under consideration those furnished by the former sex form scarcely one twelfth of the whole number. No misogynist living can

* From an extensive series of cases observed by various persons and in various quarters, M. Grisolle (p. 522,) obtains the inference that the mortality of females exceeds that of males by ⅓ or ½, that is among those actually attacked with pneumonia.

have better cause to congratulate himself on the absence of the softer sex, than M. Bouillaud on the smallness of its contributions to his pneumonic categories.

It is tolerably clear, then, that M. Bouillaud does not save more lives than his brethren. French Malthuses may dismiss the apprehensions of a glutted population, which the first accounts of the *saignées coup sur coup* might well have inspired. Even Malthus himself would scarcely have been ruffled by the elaborately tiny savings of M. Bouillaud. But M. Bouillaud is not quite baffled yet. He does not alone vaunt his method as saving a greater number of lives than any other,—" it diminishes by more than one half the duration of the disease." And how does the reader suppose this result was obtained ? In two ways. First, M. Bouillaud published a series of cases, the duration of which was estimated not from the commencement of the attack, but from the day of admission into hospital, whether this were the third, sixth, eighth, or tenth of the patient's illness ! Here was an easy receipt for the getting up of rapid cures. The cases which had lasted but about nine days, when their duration was measured *à la Bouillaud,* suddenly found their length increased to fifteen or more when estimated in the old fashioned mode. Secondly, obliged to admit the absurdity of this manner of calculation, where his advantage was gained at the *outset* of the disease, M. Bouillaud bethought him of its *close ;* and lo ! a new set of cases appears, wherein he indemnifies himself by his manner of settling the period of convalescence. This he considers to commence when the " characteristic signs of the disease and the febrile action have *almost wholly disappeared.*" Now M. Louis dates convalescence " *three days at least after the cessation of fever ;*" and yet by an unaccountable oversight (for such we must presume it to be) M. Bouillaud affirms that " he fixes the commencement of convalescence at the same time as M. Louis."

So much for the *Saignées coup sur coup.* To some other points connected with their curative omnipotence we may hereafter return. Meanwhile we congratulate M. Grisolle upon the able and uncompromising manner in which he has fathomed and exposed the depths of this most serious mischief.

Of the results obtained by the author himself we shall now give a general survey. It is necessary to premise that he divides his cases into two series,—one in which venesection was performed while the disease was in the first stage, another in which the signs of hepatization were evident when the treatment was undertaken. The first series is composed of fifty patients, who were bled from one to five times, to a mean amount of two pounds four ounces, within the first twenty-four or forty hours of their sojourn in hospital: five of these cases ended fatally. In the second series are comprised 182 patients, (143 men, 39 women,) of a mean age of thirty-five years; these subjects were bled from one to six times, and lost from eight ounces to eight pounds of blood, the mean quantity being three pounds: thirty-two of these subjects died. M. Bouillaud's cases, analysed in respect of the severity and duration of the chief symptoms after the treatment had been put in force, and compared with M. Grisolle's, furnish the plainest proof that excessive bloodletting does not ensure the least diminution in the duration of the symptoms. And it is therefore fair to proclaim, in the language of M. Grisolle, that

" M. Bouillaud having drawn blood from his patients to a mean amount of four pounds five ounces, did not obtain more favorable results in any single point of view, than I whose patients lost on an average scarcely two pounds and a half. So that it is clear M. Bouillaud caused each of his patients to lose a mean quantity of two pounds of blood without the very least utility." But is this all? Is such useless and profuse expenditure of the vital fluid to be considered merely a negation? Or is it to be esteemed positively and seriously baneful? That it is the latter no sane person can doubt.

*Tartar emetic.* We pass to the treatment of pneumonia by tartar emetic in large doses. The chapter on this subject opens with an abstract of the labours of preceding authors; this is well compiled, but the merits of Marryatt, who first prescribed the medicine in contra-stimulant doses are not mentioned: probably M. Grisolle never heard of him or them. Of the *history* of tartar emetic in its application to the treatment of pneumonia we have already spoken in this Journal, (vol. I, p. 415.) M. Grisolle's own achievements with the salt shall be our theme on the present occasion. The cases, upon which his researches bear, treated with tartar emetic in large doses, are 154 in number; he divides them into three series. A. 44 cases treated exclusively with tartar emetic, without the previous or associated use of any other mode of active medication. B. 80 cases in which the exhibition of tartar emetic was preceded by one or more general or local bleedings, these having produced no modification in the course of the disease or its principal symptoms. In none of these patients were the powers of life sufficiently sunken, to render the further loss of blood really inadmissible. C. 30 patients, who had been largely bled and nevertheless their malady was gaining ground; the state of the strength and pulse being such as to preclude the possibility of further venesection.

*Series A.* 44 patients; all seriously affected.
8 women; 36 males.
Age between 15 and 71; mean age 37.
20 of weak constitution ; 19 of medium strength; 9 vigorous.
When the treatment was commenced, the disease
had reached the stage of hepatization in 35 subjects;
was still mainly in the first stage in 9.

In all these cases the disease was of sufficiently severe character to make it impossible to predict the issue; the pulse had generally less resistance than usual, but was never of a kind formally to contraindicate bleeding. The treatment was commenced between the first and tenth days of the disease; on an average on the fourth. The quantity of the medicine taken on the first day was 6 grains, and was subsequently made to vary between 6 and 12 grains. The time it was taken varied between one and ten days, and averaged three and a half days. All these patients, without exception, and no matter what the issue of the case was destined to be, underwent the primary effects of the medicine: they had abundant stools and vomiting, of which the former predominated over the latter. Fourteen of 36 individuals, in whose cases M. Grisolle was obliged to continue the exhibition of the medicine for several days, presented a perfect state of tolerance upon the third or fourth day. Six of the 44 cases thus treated terminated fatally,—a mortality of not quite one in seven. Those patients who died were placed under more unfavorable conditions than the others; their mean age exceeded 50, the treatment was not commenced on an average until after the fifth day, and one of them had such violent delirium that the disease was rather guessed than diagnosticated during life, and the tartarized antimony administered for two days only. The duration and course of the principal symptoms under this mode of treatment are next considered; but we must pass on to

*Series B*, comprises 80 patients, whose age varied from 16 to 70, and averaged 35½. Venesection, for the most part repeated twice or thrice, had been insufficient to arrest the disease. The tartar-emetic treatment was generally commenced on the sixth or seventh day, and the medicine exhibited in the same manner as in the former series of cases, during a period varying from one to nine days, and averaging four. The mean quantity of the salt taken by each patient was 36 grains. Three patients only presented the phenomenon of tolerance from the first. Of the entire series of 80, 10 only died, a favorable result to be in part ascribed, as M. Grisolle fails not to note, to the mean age—under 36—of the patients.

In both these series of cases tartar emetic appears to have had a most obvious influence in causing rapid amelioration of the principal symptoms of the disease. In the 70 cases of recovery belonging to the series B, however, convalescence was only reestablished on the fourteenth day, that is two days later than the mean date of convalescence in patients who recovered under venesection only. A similar result was obtained by M. Louis some years since. But this result does not really prove that the medicine exercised an ill influence on the progress of the disease. We must remember that at the period tartar emetic was first administered, several bleedings had been practised without effect, the disease being on the increase in spite of them; and further, the first bleeding had been performed one day later in M. Grisolle's, two days later in M. Louis' patients treated with tartarised antimony, than in those who took none of this medicine. The result in the series A, also, distinctly disproves the charge that tartar emetic can have any influence in prolonging the duration of the malady.

*Series C.* 30 patients, of a mean age 49. The disease had already existed ten days; the possibility of further bloodletting was exhausted; the pulse soft and depressible; the patients in a state of prostration, and at least one half of them in a hopeless condition. Of these 30 patients, 18, or nearly two thirds, died; more than half of this number perished during the first two days of treatment. This result testifies, according to M. Grisolle, to the inefficacy of bleeding much more than of tartar emetic; for the former was extensively employed at an early period (on an average on the fourth day) and yet it failed in arresting the onward march of the affection.

M. Grisolle continues the subject of the tartar-emetic treatment by sections upon its modes of administration; upon its primary effects; and upon tolerance; upon the contraindications to its employment; the accidents which may follow its very abundant ingestion; and finally its *modus operandi*. The curative powers of kermes, white oxide of antimony, acetate of lead, digitalis, and hydrocyanic acid are severally discussed; and the treatment by calomel and opium dismissed with a statement that the author knows nothing of it personally, and an earnest appeal to English physicians for *proofs* of its alleged efficacy.

*Blisters.* To the *juvantia*, hygienic and medicinal, we are next conducted; and in the article on blisters find some observations with which we are disposed to tarry for a moment. It results from the analysis of 38 cases (12 fatal, 26 cases of recovery,) in which blisters were applied to the chest, that the symptoms were rarely mitigated immediately after their action, and that in the few instances in which such change occurred it was by no means certainly ascribable thereto. It was matter of demonstration that blistering did not lessen the duration of the disease. Here are precisely the opinions we have advocated in this Journal on the subject of the influence of blisters. It is just to add that M. Grisolle believes his number of cases insufficient to prove absolutely the "powerlessness and perfect inutility of blisters"—the admission we insert as a salvo for the consciences of those who, in veneration of routine, heap epispastics on every thorax they come in contact with.

But our space fails, and we find ourselves compelled to hasten to M. Grisolle's conclusion—and our own. It appears that of a total number of 304 patients observed by this physician, of a mean age of 38 years, 43 died, or something less than one in seven. Of this number of patients 80 only were submitted to the mode of treatment which M. Grisolle's analysis proves to be the best, namely, general bleeding, repeated until the pulse looses its hardness and resistance; local bleeding by leeches, or cupping, for the removal of pain; and the exhibition of tartar emetic—this medicine being commenced with before the pulse has failed to such an amount as to preclude the possibility of further bloodletting. We believe that this is fundamentally and mainly the practice of the enlightened physicians of Great Britain at the present day. "The care," observes M. Grisolle, in his closing words, "with which I have studied the question of treatment gives me motive for hope that I have not erred; and I think I may affirm, in the language of Sydenham, that the treatment I recommend is that which I should wish applied in my own case, were I seized with the malady of which I have now traced the history."

It is impossible to have studied M. Grisolle's volume without feeling strong admiration for the industry and sound judgment exhibited in its composition. The author cannot, it is true, lay claim to the discovery of any striking novelty; but he may congratulate himself on having performed a far more useful task than the promulgation of a few new notions. He has accumulated the experiences of his predecessors, subjected them to a searching and impartial criticism, separated the true from the false, substantiated the solid, and scattered to the winds many empty opinions of contemporary observers by a logical appreciation of his own experience. All this he has done with unswerving good faith. If in closing the volume we must, not to lose our reputation for good critics, cavil, we would express our regret that the works of English writers have not been more carefully studied in the *originals*. M. Grisolle, for example, appears to know the treatise of Dr. Stokes but by extracts in the Parisian journals—yet even thus known he finds in it just theme for praise. If garbled fragments, often ill selected and carelessly translated, are rife with the most valuable information, the importance of mastering the entire treatise should surely not have been overlooked by M. Grisolle. Further, the volume we have reviewed is so monstrously prolix that its readers must be limited to the Herculeses of literature alone—and to the professional critic. But as a book of reference, especially as illustrative of French opinion, the work cannot be pronounced less than invaluable.

